# 2606. Epidemiology of invasive pneumococcal disease in adults with and without underlying lung disease in Ontario, Canada

**DOI:** 10.1093/ofid/ofad500.2220

**Published:** 2023-11-27

**Authors:** Zoe Zhong, Altynay Shigayeva, Agron Plevneshi, Irene Martin, Walter Demczuk, Julianne Kus, Mahin Baqi, Danny Chen, Wayne Gold, Reena Lovinsky, Neil Rau, David Richardson, Allison McGeer

**Affiliations:** Sinai Health System, University of Toronto, Toronto, Ontario, Canada; Sinai health, Toronto, Ontario, Canada; Sinai Health System, Toronto, Ontario, Canada; National Microbiology Laboratory (NML), Winnipeg, MB, Canada; Public Health Agency of Canada, Winnipeg, Manitoba, Canada; Public Health Ontario, Toronto, Ontario, Canada; William Osler Health System, Toront, Ontario, Canada; MacKenzie Health, Vaughan, Ontario, Canada; University of Toronto, Toronto, ON, Canada; Scarborough Health Network, Toronto, Ontario, Canada; Halton Healthcare, Oakville, Ontario, Canada; William Osler Health System, Toront, Ontario, Canada; Mt. Sinai Hospital, Toronto, Ontario, Canada

## Abstract

**Background:**

The advent of enhanced conjugate pneumococcal vaccines for adults has created a need to assess the burden of pneumococcal disease in different populations to support recommendations for vaccination. We assessed the epidemiology of invasive pneumococcal disease (IPD) in adults with underlying lung disease in Ontario, Canada.

**Methods:**

Ontario introduced PCV7 and PCV13 for children in 2007 and 2010, and PCV13 for immunocompromised adults over 50 years in 2014. TIBDN performs population (pop’n)-based surveillance for IPD in a pop’n of ∼4.5 million. Microbiology laboratories serving area residents report sterile site isolates of *Streptococcus pneumoniae*; annual audits ensure completeness. Isolates are serotyped at the National Microbiology Laboratory. Population data are from Statistics Canada, with estimates of prevalence of underlying conditions from ICES.

**Results:**

From 2014-2022, 2769 episodes of IPD occurred in adults; detailed clinical data are available for 2366 (85%), and serotyped isolates for 88%. Of these, 573 (24%) had no chronic underlying illness (undill); 1155 (49%) had an undill not affecting the lungs, 165 (7%) had asthma only, 409 (17%) had COPD (with/without asthma or other lung disease (OLD), 64 (2.7%) had OLD alone (most commonly ILD or bronchiectasis). Among those with asthma, 87 (53%) had another undill predisposing to IPD; compared to 290 (71%) of those with COPD and 45 (70%) of those with OLD. Persons with asthma were younger (median age 56y) than those with no or other undill (64y and 61y), and those with COPD or OLD (70 and 71y). Persons with lung disease were more likely to present with bacteremic pneumonia and less likely to have primary bacteremia or meningitis that those without or with other chrill. ICU admission and case fatality rates are in Table 1. The distribution of serotypes causing disease by conjugate vaccine types did not differ among those with lung disease and others (Figure 1). The incidence of disease in persons with asthma and COPD by age group is shown in Figure 2.

**Figure:**
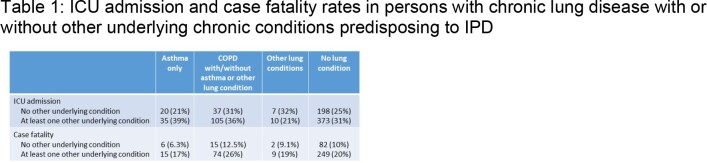

**Figure:**
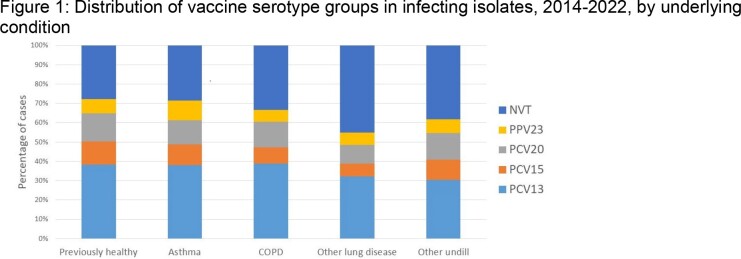

**Figure:**
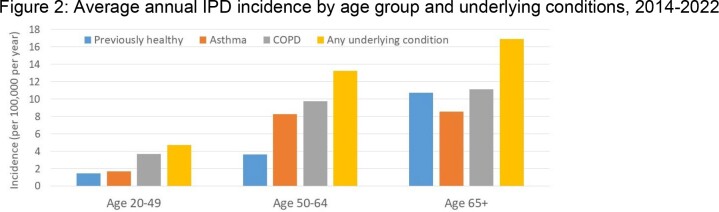

**Conclusion:**

The epidemiology of IPD differs somewhat in persons with lung disease compared to others, but conditions other than lung disease have a greater impact on incidence and outcome of IPD in adults. Enhanced PCVs likely provide cost-effective protection for 50-64 year olds with asthma/COPD.

**Disclosures:**

**All Authors**: No reported disclosures

